# ﻿Complete mitochondrial genomes of *Sinonovacularivularis* and *Novaculinachinensis* and their phylogenetic relationships within family Pharidae

**DOI:** 10.3897/zookeys.1232.139844

**Published:** 2025-03-19

**Authors:** Yiping Meng, Liyuan Lv, Zhihua Lin, Demin Zhang, Yinghui Dong

**Affiliations:** 1 School of Marine Sciences, Ningbo University, Ningbo 315010, China Ningbo University Ningbo China; 2 College of Advanced Agricultural Sciences, Zhejiang Wanli University, Ningbo 315101, China Zhejiang Wanli University Ningbo China; 3 Ninghai Institute of Mariculture Breeding and Seed Industry, Zhejiang Wanli University, Ninghai 315604, China Zhejiang Wanli University Ninghai China

**Keywords:** Gene arrangement, mitogenome, Pharidae, phylogeny, positive selection

## Abstract

Pharidae is one of the most ecologically and commercially significant families of marine Bivalvia; however, the taxonomy and phylogeny of Pharidae has been ongoing for quite some time and remains a contentious issue. Here, to resolve some problematical relationships among this family, the complete mitochondrial genomes (mitogenomes) of *Sinonovacularivularis* (17,159 bp) and *Novaculinachinensis* (15,957 bp) were assembled, and a comparative mitochondrial genomic analysis was conducted. Both mitogenomes contain 12 protein-coding genes, 22 transfer RNA genes, and two ribosomal RNA genes. Among the published Pharidae mitogenomes, *N.chinensis* exhibited the smallest genome size but the highest AT content. The results of the phylogenetic trees confirmed the monophyly of the family Solenoidea, and indicated that *N.chinensis* and *Sinonovacula* (*S.constricta* and *S.rivularis*) were closely related in the family Pharidae. From the CREx analysis, we found that transposition and tandem duplication random losses (TDRLs) might have occurred between Pharidae and Solenidae. Moreover, positive selection was detected in nad5 of the foreground *N.chinensis*, and divergent evolution occurred at site 144 in the freshwater and marine lineages. Overall, our findings provide new molecular data on the phylogenetic and evolutionary relationships of Pharidae, and contribute to unraveling the salinity adaptations of Pharidae.

## ﻿Introduction

Pharidae belongs to Solenoidea which is one of the most ecologically and commercially significant superfamilies of marine Bivalvia, and the North-West and Indo-West Pacific regions exhibit the highest levels of species diversity, encompassing approximately 85% of all species, predominantly distributed in intertidal zones (Lin 2009; Saeedi et al. 2016; Costello and Saeedi 2019). According to the China Fisheries Statistics Yearbook (2024), the annual output of razor clams is 850,000 tons, accounting for 5.16% of the total output of mollusks. This Pharidae family has an extensive fossil record, dating back to approximately 103 million years ago (Mya) in the middle Cretaceous (Bolotov et al. 2018b). Although Pharidae is well-established as a clade, the internal taxonomic research has been ongoing and remains a contentious problem. Cosel (1993) promoted Solenidae to Solenoidea in 1993 and divided the superfamily into Solenidae and Pharidae according to the number of main teeth. Among them, the genus *Sinonovacula* was once classified by Graham into the family Solecurtidae, which belongs to the superfamily Tellinoidea (Graham 1935). However, an increasing number research findings contradict this, where the genus *Sinonovacula* should be categorized into the family Pharidae (Taylor et al. 2007; Guoquan et al. 2010; Yuan et al. 2012c; Yu et al. 2016). For example, the comparison of mitogenomes of six heterodont bivalves indicated that *S.constricta* (Lamarck, 1818) was more closely related to *Solengrandis* (Dunker, 1862), which belonged to Solenidae (Yuan et al. 2012c). The phylogenetic tree and molecular clock of tandem mitochondrial gene and nuclear gene (*COI*, *16S*, *28S*) revealed that *Siliqua*, *Sinonovacula*, *Cultellus*, and *Novaculina* belonged to Pharellinae, and *Pharellajavanica* (Lamarck, 1818) was classified under the *Sinonovacula* subclade (Bolotov et al. 2018b). Moreover, Pharidae were divided into four subfamilies which were composed of 14 existing genera, including Pharinae (*Nasopharus*, *Pharus*, *Sinupharus*), Cultellinae (*Afrophaxas*, *Cultellus*, *Ensis*, *Ensiculus*, *Phaxas*, *Sinucultellus*), Siliquinae (*Siliqua*), and Pharellinae (*Novaculina*, *Orbicularia*, *Pharella*, *Sinonovacula*), and Bolotov et al. (2018b) argued that Novaculininae was considered to be a junior synonym of Pharellinae (Appeltans et al. 2012; Signorelli et al. 2021). Nevertheless, since the above studies are only based on a limited number of taxa, the phylogenetic relationship of Pharidae has not been fully studied.

Mitochondrial DNA (mtDNA) is a genetic material independent of the nucleus DNA. Owing to their small size, rapid evolution, maternal inheritance, and simple structure, mitogenomes have become an attractive candidate tool for resolving phylogenetic relationships across a wide spectrum of metazoans (Boore 1999; Curole and Kocher 1999; Saccone et al. 1999; Miya et al. 2001; Gissi et al. 2008; Osigus et al. 2013; Cameron 2014). Mitogenomes of metazoan are usually circular double-stranded molecules, and range in size from 14 kb to 42 kb (Okimoto et al. 1992; Wolstenholme 1992; Smith and Snyder 2007). The typical mitogenome is composed of 37 genes compactly organized in a near-invariant arrangement, including 13 protein-coding genes of the oxidative phosphorylation (OXPHOS) system (*cox1–3*, *cob*, *nad1–6*, *nad4L*, *atp6*, *atp8*), 22 transfer RNAs (tRNAs) and two ribosomal RNAs homologous to the 16s and 23s of *Escherichiacoli* (*rrnS* and *rrnL*) (Wolstenholme 1992; Shadel and Clayton 1997; Andrews et al. 1999; Boore 1999). In general, metazoan mtDNA molecules have few or no nucleotides between genes except for a single non-coding region that contains signals for regulating replication and transcription (designated as the control region) (Clayton 1984; Wolstenholme 1992; Shadel and Clayton 1997). However, the phylum Mollusca has generated a vast array of unexpected deviations from the ‘textbook description’, including exceptional variation in size, frequent genome rearrangements, the integration of novel genes, and a complex inheritance system dubbed ‘doubly uniparental inheritance’ (Wu et al. 2012; Williams et al. 2017; Wu et al. 2019; Malkócs et al. 2022).

In mollusks, with the development of DNA sequencing technology, a large number of mitogenomes have been determined during the last thirty years (Yokobori et al. 2004; Yuan et al. 2012d; Kong et al. 2020; Ma et al. 2023; Taite et al. 2023). For instance, through comparing the complete mtDNA sequences of three scallop species from the subfamily Chlamydinae, it was found that the three genomes exhibited high variation in non-coding regions and different tRNA gene sets (Wu et al. 2009). Besides, the results of the phylogenetic analysis based on concatenated 12 protein-coding genes (PCGs) and two rRNA genes validated the monophyly of the family Mactridae and indicated that genera *Mactrinula*, *Spisula*, *Rangia*, and *Mulinia* should not be placed under subfamily Mactrinae (Ma et al. 2023). Nevertheless, to date, only four mitogenomes of Pharidae, which are ecologically and economically important deep-burrowing bivalves, are available (Zheng et al. 2010; Feng et al. 2021; Li et al. 2022).

*Sinonovacularivularis* (R. Huang & Y.-F. Zhang, 2007), the member of the genus *Sinonovacula*, is similar to *S.constricta* in reproduction and morphology (Huang and Zhang 2007). In contrast to *S.constricta*, which exhibits tolerance to wide salinity (5–40 ppt), *S.rivularis* is capable of thriving in low salt aquatic environments (4–20 ppt), and can even endure in freshwater conditions for over four days (Huang and Zhang 2007; Wang et al. 2009; Peng et al. 2019; Wang et al. 2024). In addition, a typical freshwater genus *Novaculina* is found in the family Pharidae (Schram 2010; Bolotov et al. 2018b). As a species of *Novaculina*, *N.chinensis* (Y.-Y. Liu & W.-Z. Zhang, 1979) was first discovered in Taihu Lake and Gaoyou Lake in China (Liu 1979). However, due to the pollution of water and the lack of protection awareness, they have been in danger of extinction (Liu 1979; Rao et al. 2003). In this study, we assembled the complete mitogenome of *S.rivularis* and *N.chinensis*, and analyzed their basic genome characteristics, nucleotide composition and relative synonymous codon usage (RSCU). The phylogenetic tree of Solenoidea was constructed and gene arrangement events between Pharidae and Solenidae were predicted. Furthermore, selective pressure analysis was conducted to explore the evolutionary adaptation of freshwater and marine species. Briefly, our findings will enrich the basis for the taxonomic study of Pharidae and contribute to deepening the understanding of the phylogenetic relationship between Solenoidea and its related groups.

## ﻿Materials and methods

### ﻿Sample collection

The samples for whole-genome sequencing of *S.rivularis* and *N.chinensis* were collected from the coastal area of Quanzhou in Fujian Province and the Qiantang River in Zhejiang Province, respectively, following the relevant guidelines and regulations. A total of ten individuals each of *S.rivularis* and *N.chinensis* were sampled, with average shell length of 55.98 ± 3.47 mm and 45.41 ± 2.74 mm, respectively. All specimens were preserved in 85% ethanol as voucher specimens. These specimens were deposited at Zhejiang Key Laboratory of Aquatic Germplasm Resource, Zhejiang Wanli University, Ningbo, China.

### ﻿Mitogenome assembly and annotation

Raw genome reads were acquired through both Illumina HiSeq sequencing and PacBio Sequel IIe third-generation sequencing (unpublished), and assembled for the mitogenomes of these two species. Initially, a de novo mitogenome assembly was carried out with SPAdes v3.9.0 after filtering the unqualified reads by Trimmomatic v. 0.39 (Bankevich et al. 2012; Bolger et al. 2014). The scaffold sequences were then obtained by extending the contigs using SSPACE. The assembly quality was evaluated by GetOrganelle software (Jin et al. 2020). Finally, the MitoZ program was used to annotate the protein-coding genes (PCGs), two ribosomal RNAs (rRNAs) and transfer RNAs (tRNAs) (Meng et al. 2019).

### ﻿Mitogenome characteristics analysis

The content and proportion of nucleotide bases were analyzed by MEGA 11. The base skew values were calculated according to the formulae: AT-skew = (A − T) / (A + T) and GC-skew = (G − C) / (G + C). The RSCU of the two mitogenomes was counted using PhyloSuite v1.2.3.

### ﻿Phylogenetic analysis and gene arrangement analysis

To explore the evolutionary relationship of *S.rivularis* and *N.chinensis*, the published mitogenome sequences of Solenoidea and Hiatelloidea were retrieved from GenBank, and *Solecurtusdivaricatus* was selected as the outgroup (Table 1). The phylogenetic analysis was performed using PhyloSuite software (Zhang et al. 2020). First, using an invertebrate mitochondrial code table, MAFFT was used to independently align 12 protein-coding genes. The *ATP8* gene was excluded due to its deletion in the majority of mollusks. Poorly aligned regions of the sequences were pruned by Gblocks under default parameters. The resulting alignments were then concatenated and transferred to ModelFinder for the best model prediction. Phylogenetic trees were estimated through maximum likelihood (ML) and Bayesian inference (BI) methods. The ML phylogenetic tree was generated using IQ-Tree with 1000 bootstrap replicates. The BI analyses were performed by MrBayes 3.2.6 with Markov Chain Monte Carlo (MCMC) for 5000,000 generations. The first 25% of trees were discarded as burn-in and the sampling was terminated when the convergence value was less than 0.01. The iTOL tool was exploited to visualize the phylogenetic tree (https://itol.embl.de/).

**Table 1.** List of species used for phylogenetic analysis in this study and their GenBank accession numbers.

Order	Superfamily	Family	Species	Length (bp)	Accession number	Percent of AT (%)
Adapedonta	Solenoidea	Pharidae	*Novaculinachinensis*	15,957	PP874232	71.50
			*Sinonovacularivularis*	17,159	PP874231	66.80
			*Sinonovaculaconstricta*	17,224	JN398366.1	67.00
			*Ensisleei*	16,926	MW727513.1	65.50
			*Cultellusattenuatus*	16,888	MW653805.1	66.46
			*Siliquaminima*	17,064	MT375556.1	66.41
		Solenidae	*Solenstrictus*	16,535	NC_017616.1	62.70
			*Solengrandis*	16,784	NC_016665.1	64.84
	Hiatelloidea	Hiatellidae	*Panopeaabrupta*	15,381	NC_033538.1	64.40
			*Panopeaglobosa*	15,469	NC_025636.1	63.70
			*Panopeagenerosa*	15,585	NC_025635.1	63.70
			*Panopeajaponica*	16,128	NC_072278.1	63.80
			*Hiatella* sp.	19,507	OR420023.1	64.00
			*Hiatellaarctica*	18,244	DQ632742.1	66.40
Cardiida	Tellinoidea	Solecurtidae	*Solecurtusdivaricatus*	16,749	JN398367.1	60.10

In addition, the most plausible gene order rearrangement events that might have occurred between Pharidae and Solenidae were reconstructed by pairwise comparisons of mitogenomes through the Common Interval Rearrangement Explorer (CREx) (Bernt et al. 2007).

### ﻿Selective pressure analysis

The branch-site model was used to analyze the selection pressure on 12 PCGs of razor clams in the PAML package. In this model, *N.chinensis* was marked as the foreground branch to investigate the evolutionary adaptation between freshwater and marine species. The null model (model = 2, Nssites = 2, fix_omega = 1, omega = 1) and alternative model (model = 2, Nssites = 2, fix_omega = 0, omega = 2) were compared by likelihood ratio test (LRT). Subsequently, *P*-values were calculated through the chi-square distribution. Then, the posterior probability of the amino acid sites under positive selection was calculated according to the Bayesian empirical Bayes (BEB) method. The inference of positively selected sites was based on a posterior probability of greater than 95%.

## ﻿Results

### ﻿General features of *S.rivularis* and *N.chinensis* mitogenomes

**Figure 1.**
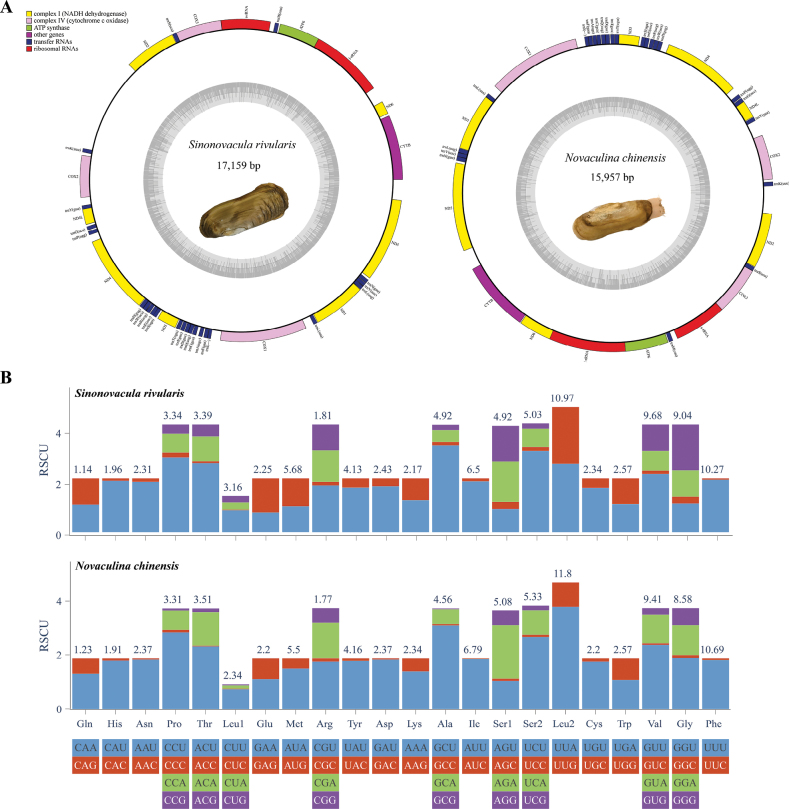
Maps of **A** the mitogenomes of *S.rivularis* and *N.chinensis* and their **B**RSCU.

The lengths of *S.rivularis* and *N.chinensis* mitogenomes were 17,159 bp and 15,957 bp, respectively (Fig. 1A). Both mitogenomes contain 12 PCGs, 22 tRNAs, and 2 rRNAs, all of which were located on the heavy chain. The *ATP8* gene was missing in this two mitogenomes. Their composition was similar to that of other species in Pharidae, indicating a certain degree of conservation in this family. The detailed genes information was shown in Table 2. The base composition of *S.rivularis* and *N.chinensis* mitogenomes was displayed in Table 3 with AT contents of 66.80% and 71.50%, respectively, both of which exhibited an obvious AT bias. The AT content of *N.chinensis* was the highest among the published Adapedonta mitogenomes. In addition, the two mitogenomes all exhibited negative AT-skew and positive GC-skew, reflecting that the base composition ratios were A biased to T, and G biased to C. There were some differences in the types of start and termination codons of 12 PCGs between the two species (Table 2). Specifically, the start codons of 12 genes in *S.rivularis* were found to be ATN, TTG and GTG types, whereas in *N.chinensis*, all genes began with the codon ATN, with the exception of the *ND4* gene, which used TTG as the start codon. Concerning termination codons, six genes in *S.rivularis* (*cytb*, *atp6*, *cox3*, *nad4*, *nad3*, *nad1*) and seven genes in *N.chinensis* (*cytb*, *nad6*, *atp6*, *cox3*, *nad4l*, *nad3*, *nad1*) were detected TAA or TAG at the sequence end. The remaining genes featured an incomplete termination codon consisting of a T that might be complemented into a complete stop codon by polyadenylation following transcription to the resultant mRNA (Ojala et al. 1981). Furthermore, the non-coding regions of the mitochondrial genomes of *N.chinensis* and *S.rivularis* account for 11.92% and 19.33%, respectively. The longest non-coding region (NCR) of *N.chinensis* and *S.rivularis* was both located between nad2 and trnK, with lengths of 443 bp and 1,639 bp respectively, which was identified as a putative control region (CR).

**Table 2.** Mitochondrial genome organization of *Sinonovacularivularis* and *Novaculinachinensis*.

Gene	*Sinonovacularivularis*					*Novaculinachinensis*				
	Size (bp)	Start	End	Codon start/stop	Intergenic nucleotide (bp)	Size (bp)	Start	End	Codon start/stop	Intergenic nucleotide (bp)
*CYTB*	1120	13	1132	TTG/TAG	36	1146	9247	10392	ATG/TAA	12
*ND6*	227	1169	1395	TTG/T--	265	531	10405	10935	ATG/TAG	-30
*l-rRNA*		1661	2957		-35		10906	12201		
*ATP6*	700	2923	3622	ATG/TAA	23	699	12164	12862	ATG/TAA	15
*trnM*		3646	3713		76		12878	12943		77
*s-rRNA*		3790	4637		-2		13021	13869		-2
*COX3*	790	4636	5425	ATG/TAG	-2	789	13868	14656	ATG/TAG	-1
*trnS*		5424	5491		5		14656	14722		6
*ND2*	899	5497	6395	GTG/T--	1639	898	14729	15626	ATT/T--	443
*trnK*		8035	8102		48		113	179		45
*COX2*	725	8151	8875	ATG/T--	132	726	225	950	ATG/T--	256
*trnY*		9008	9072		-20		1207	1270		7
*ND4L*	287	9053	9339	ATT/T--	33	288	1278	1565	ATG/TAA	1
*trnG*		9373	9439		20		1567	1632		10
*trnP*		9460	9525		122		1643	1707		122
*ND4*	1354	9648	11001	TTG/TAG	8	1254	1830	3083	TTG/T--	103
*trnH*		11010	11076		-1		3187	3250		2
*trnW*		11076	11144		2		3253	3319		3
*trnR*		11147	11213		13		3323	3387		18
*trnE*		11227	11294		-7		3406	3472		-6
*trnS*		11288	11351		30		3467	3529		33
*ND3*	337	11382	11718	ATA/TAA	15	333	3563	3895	ATT/TAG	-1
*trnT*		11734	11800		9		3895	3960		3
*trnI*		11810	11876		8		3964	4029		15
*trnD*		11885	11951		-1		4045	4110		0
*trnQ*		11951	12018		5		4111	4178		10
*trnC*		12024	12092		42		4189	4253		2
*trnA*		12135	12200		23		4256	4320		5
*trnF*		12224	12288		223		4326	4389		200
*COX1*	1488	12512	13999	CGA/**T**--	142	1512	4590	6101	ATT/T--	154
*trnL*		14142	14209		8		6256	6320		0
*ND1*	919	14218	15136	GTG/TAA	2	927	6321	7247	ATG/TAA	4
*trnL*		15139	15207		1		7252	7319		0
*trnV*		15209	15274		2		7320	7383		0
*trnN*		15277	15343		35		7384	7449		36
*ND5*	1443	15379	16821	ATT/T--	350	1441	7486	8926	ATT/**T**--	320

**Table 3.** Nucleotide composition and skewness of the mitogenomes of *S.constricta*, *S.rivularis*, and *N.chinensis*.

Species	AT (%)	GC (%)	AT skew	GC skew
*S.constricta*	67.00%	32.90	-0.22687	0.367781
*S.rivularis*	66.80%	28.50	-0.21958	0.319298
*N.chinensis*	71.50%	33.20	-0.23653	0.379518

As illustrated in Fig. 1B, the preferred codons for 22 amino acids of two species ended in A or U, consisting with the result of AT bias of the mitogenome sequence. As a consequence of the duplication of tRNA-Leu and tRNA-Ser, Leu and Ser were each encoded by six and eight codons, respectively. The most frequently used codons were UUA (Leu2), UCU (Ser2), GCU (Ala) and CCU (Pro). Compared to *S.rivularis*, CUG (Leu1), AUC (Ile), AAC(Asn) were utilized to a lesser extent in *N.chinensis*.

### ﻿Phylogenetic analysis

**Figure 2.**
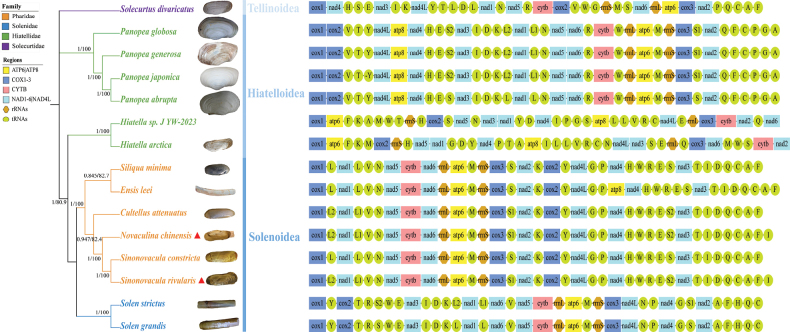
The phylogenetic trees based on concatenated 12 mitochondrial PCGs, and the gene orders of Adapedonta species. Values shown next to nodes are posterior probabilities (left) and ML bootstrap support values (right). Newly assembly mitogenomes are marked with triangles. Except for *Panopeaabrupta* (https://inverts.wallawalla.edu) and *Panopeaglobosa* (Góngora-Gómez et al. 2016), the images of the other species are all from https://www.inaturalist.org.

The 12 protein-coding genes from 15 taxa were concatenated to generate a sequence matrix of 10,806 bp. The tree topologies derived from the ML and BI analyses were largely congruent exhibiting high posterior probabilities (PP) and bootstrap support values (BS) in most nodes (Fig. 2). Phylogenetic analyses revealed that the genus *Hiatella* from Hiatelloidea was closely related to the superfamily Solenoidea, indicating a close evolutionary relationship between them. Additionally, both analyses strongly confirmed the monophyly of Solenoidea, which was divided into two major branches, Solenidae and Pharidae. In the family Pharidae, the genus *Sinonovacula* (including *S.rivularis* and *S.constricta*) was clustered alongside *N.chinensis*, with *Cultellusattenuatus* emerging as a sister group. *Siliquaminima* and *Ensisleei* were clustered in a separate cluster.

### ﻿Gene arrangement

**Figure 3.**
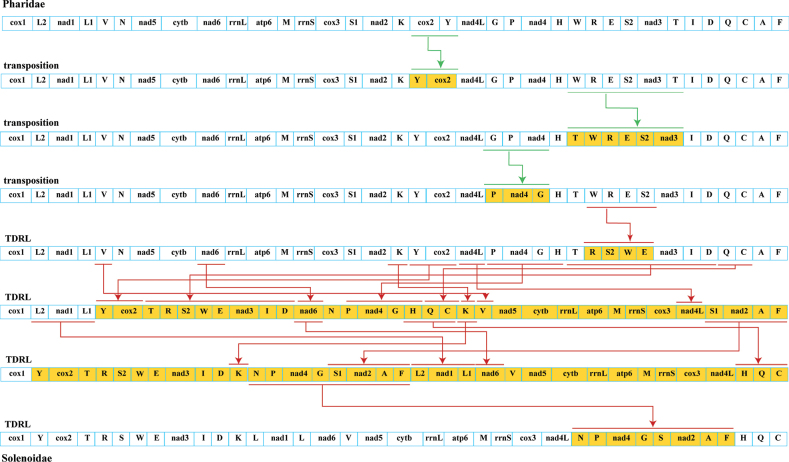
Putative gene rearrangement events between Pharidae and Solenidae. Green and red lines represent transposition and TDRL events, respectively, which were step by step identified by CREx.

The mitogenomes of Solenoidea all exhibited the identical composition of 12 PCGs, 22 tRNAs, and 2 rRNAs, except for *Ensisleei*, which contained an additional *ATP8* gene (Fig. 3). The gene arrangement was consistent within each family, and there was a certain level of conservation in gene arrangement between Solenidae and Pharidae. A large block, rrnL-ATP6-M-rrnS-cox3, and five small blocks, L2-nad1-L1, S-nad2, nad5-cytb, I-D, Q-C were shared by both families, providing further evidence of the close lineage relationship observed in the phylogenetic analysis of this study. The CREx analysis suggested that three transposition and four tandem duplication random losses (TDRLs) might have occurred between Pharidae and Solenidae.

### ﻿Select pressure analysis

**Figure 4.**
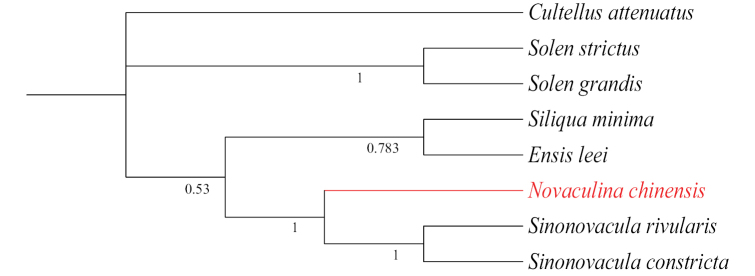
Phylogenetic tree of Solenoidea for selective stress analysis. The branch marked in red is the foreground branch.

**Figure 5.**
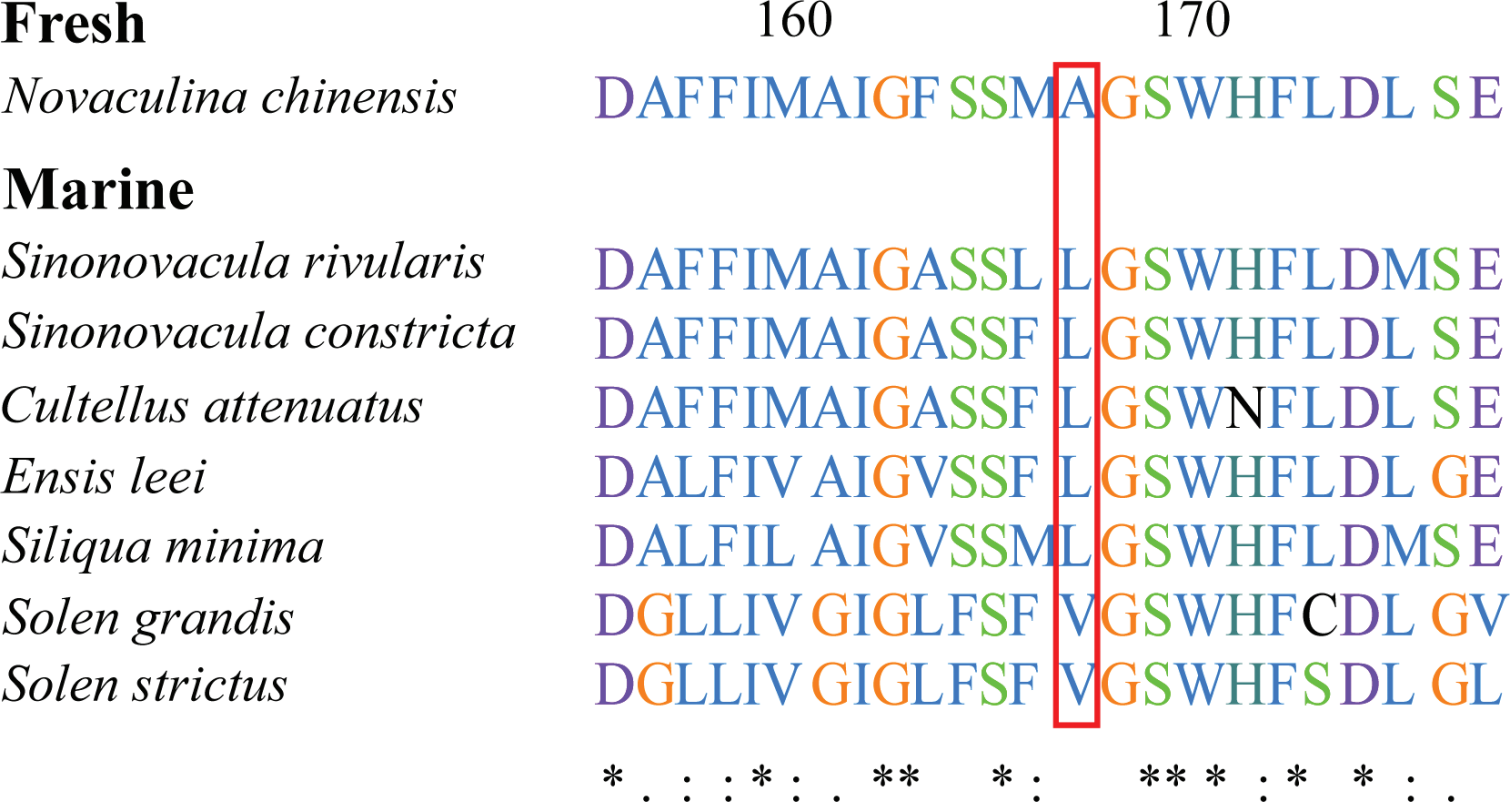
The difference of the 144^th^ positive selected amino acid site in NAD5 of eight Solenoidea species. The 144^th^ site is indicated by a red frame.

The species of Solenoidea were selected for molecular evolution analysis, with *N.chinensis* designated as the foreground branch (Fig. 4). The branch-site model (BSM) in the PAML package was employed to detect positively selected genes (PSGs). As illustrated in Table 4, the substitution model A was significantly better than the neutral selection model null in *nad5*, indicating that this gene underwent positive selection in the foreground branch (P < 0.05). According to the BEB analysis, there were five positive selection sites in the nad5 amino acid sequences (140 A 0.509, 143 F 0.547, 144 L 0.865, 442 A 0.700, 446 F 0.620). Moreover, discrepancies were observed in the 144^th^ site between freshwater *N.chinensis* (Ala) and marine razor clams (Leu) (Fig. 5). However, the evidence for each site was somewhat inconclusive. These findings suggest that the *nad5* gene may have played a pivotal role in the adaptive evolution of freshwater environments.

**Table 4.** The results of positively selected gene sites for 12 PCGs.

Gene	lnL0	lnL1	Np0	Np1	Omega	P value	Positively selected sites (PSGs)
*nad3*	-1552.22	-1552.22	17	18	2.52856	1	
*nad1*	-4134.56	-4134.56	17	18	2.35774	1	
*cytb*	-4826.79	-4826.79	17	18	2.62875	1	
*nad4L*	-1236.77	-1236.78	17	18	3.31711	0.895254	
*nad5*	-6913.41	-6842.58	17	18	3.34388	0	140 A 0.509, 143 F 0.547, 144 L 0.865, 442 A 0.700, 446 F 0.620
*cox1*	-5205.94	-5205.94	17	18	2.64645	0.998872	
*nad2*	-4693.2	-4693.2	17	18	3.07094	1	
*nad6*	-877.007	-877.007	17	18	2.68959	1	
*nad4*	-6088.71	-6088.71	17	18	3.25046	1	
*cox3*	-3173.94	-3173.94	17	18	2.39696	1	
*cox2*	-2763.71	-2763.71	17	18	1.96195	1	
*atp6*	-2882.5	-2884.28	17	18	3.30394	0.058789	

## ﻿Discussion

### ﻿General features of Pharidae mitogenomes

The mitogenomes of *S.rivularis* and *N.chinensis* were newly assembled, with lengths of 17,159 and 15,957 bp, respectively. In compared with the previously sequenced AdapedontamtDNA size (ranged from 15,381 bp to 19,507 bp), their mitogenome sizes were within the normal range (Zheng et al. 2010; Yuan et al. 2012b; Feng et al. 2021; Li et al. 2022). Notably, the genome size of *N.chinensis* was the smallest in the family Pharidae, which was associated with the variation in length of the control region. The CR is the region with the largest sequence and length variation in the mitogenome, and has the fastest evolution, which is crucial for the regulation of mitochondrial DNA replication and transcription (Wolstenholme 1992; Boore 1999). The substantial differences in the content and structure of the control region within the mollusk lineage provide valuable insights for population genetic analysis (Sasuga et al. 1999; Tomita et al. 2002; Kawashima et al. 2013). Among the published mitogenomes of Pharidae, there is a large control region between *nad2* and *trnK*, such as *S.constricta* (1,492 bp), *S.minima* (1,371 bp), *C.attenuatus* (1,173 bp) and *E.leei* (1,101 bp) (Zheng et al. 2010; Feng et al. 2021; Li et al. 2022). In this study, *S.rivularis* displayed a moderately larger control region size of 1,639 bp, whereas it was only 441 bp in *N.chinensis*, making it a different mitogenome size in the family Pharidae. Intriguingly, a similar control region was not observed in the species of Solenidae (Yuan et al. 2012a, b). This distinction provides evidence for the taxonomic division of the subfamily of Solenoidea.

### ﻿Molecular phylogeny and gene arrangement of the family Pharidae

The topological tree constructed from the 12 mitochondrial PCGs sequence based on the BI and ML methods yielded consistent results, demonstrating that Solenoidea is clearly divided into Solenidae and Pharidae, which is consistent with the prior research results (Yuan et al. 2012d; Feng et al. 2021). Previously, *S.rivularis* was identified as a new species of *Sinonovacula* distinct from *S.constricta* based on morphological studies and a comparative analysis of COI and 16SrRNA fragments (Huang and Zhang 2007; Weng et al. 2013). In this research, this classification view was supported at the level of mitogenomes, and *Sinonovacula* belonged to the family Pharidae (Adapedonta: Solenoidea). In addition, *N.chinensis* was previously classified into Solecurtidae, whereas the results of this study demonstrated that *N.chinensis* and *Sinonovacula* are clustered together, forming a novel branch in the family Pharidae, which was consistent with the taxa in WoRMS (Liu 1979; Appeltans et al. 2012). Recently, the phylogenetic tree and molecular clock of tandem mitochondrial gene and nuclear gene (*COI*, *16S*, *28S*) revealed that *Siliqua*, *Sinonovacula*, *Cultellus*, and *Novaculina* belonged to Pharellinae (Bolotov et al. 2018b). However, *Cultellus* and *Siliqua* were categorized into the subfamily Cultellinae and Siliquinae, respectively, by Ahyong (Appeltans et al. 2012). Previously, Pharidae were divided into four subfamilies: Pharinae (*Nasopharus*, *Pharus*, *Sinupharus*), Cultellinae (*Afrophaxas*, *Cultellus*, *Ensis*, *Ensiculus*, *Phaxas*, *Sinucultellus*), Siliquinae (*Siliqua*), and Pharellinae (*Novaculina*, *Orbicularia*, *Pharella*, *Sinonovacula*). However, the present results indicate that Pharidae are divided into two clades, in which *Cultellus* is clustered alongside the *Sinonovacula* and *Novaculina*, while *Siliqua* and *Ensis* clustered together. These observations reflect that the current categorization of the subfamily Pharidae requires further research and refinement, particularly in combination with more species information.

Unlike stable gene arrangements of Vertebrata and Arthropoda, the gene orders of all genes within mtDNA exhibit considerable variability in every major molluscan lineage, including Cephalopoda, Bivalvia, Scaphopoda, and Monoplacophora (Rawlings et al. 2001; Dreyer and Steiner 2004; Yuan et al. 2012c; Stöger et al. 2016; Ma et al. 2023). Gene rearrangements may be caused by reverse transpositions, transpositions, inversions, and TDRL, which can provide important clues about the evolutionary history of species (Boore and Brown 1998; Serb and Lydeard 2003; Wang et al. 2021). In this paper, CREx analysis predicted that three transpositions and four TDRLs might have occurred between Pharidae and Solenidae, implying that dramatic mitogenome changes occurred during species differentiation. Moreover, the gene order illustration of Adapedonta revealed that species with a closer genetic relationship tended to share a similar gene arrangement, indicating that there is a potential relationship between evolution and gene rearrangement (Fig. 2). However, in this study, three distinct gene arrangement types were observed in the family Hiatellidae, especially in the genus *Hiatella* with nad3 and nad1 transpositions in terms of 12 PCGs arrangement (Fig. 2). The similar case that different gene arrangements in the same genus has also been reported in the genera *Dendropoma* and *Crassostrea* (Rawlings et al. 2010; Ren et al. 2010). Therefore, the taxonomic evolution of species cannot be substantiated exclusively through the examination of gene sequences; it also necessitates the integration of phylogenetic reconstruction.

### ﻿Adaptive evolution of Pharidae mitochondrial genes to freshwater environment

Pharidae is a major marine family, with the exception of *Novaculina*, that is a relict marine-derived freshwater lineage (Annandale 1922; Bolotov et al. 2018a). The branch-site model study was used to determine whether positive selection occurs at a few places in freshwater razor clam. The results suggested that the *nad5* gene underwent positive selection. NADH dehydrogenase is the initial and most substantial enzyme complex in the respiratory chain, functioning as a proton pump (da Fonseca et al. 2008). *Nad2*, *nad4*, and *nad5* are considered to be the actual proton pumping devices because of their sequence homology with a class of Na^+^/H^+^ antiporters (Brandt 2006). The efficiency of the proton transfer process may be interfered by the mutation of the complex, which could be a crucial factor in adaptive evolution (Hassanin et al. 2009; Yu et al. 2011). For instance, the outcomes of positive selection sites in mussels from disparate habitats reflected that the p-value of *nad4* was significant in freshwater branches and six sites were identified as positive sites with BEB analysis (> 95%), which implies that *nad4* may contribute to the adaptation of *Limnopernafortunei* in freshwater (Zhao et al. 2022). Significant non-synonymous changes were detected in the *cytb* and *nad5* genes by comparing mitogenomes of panpulmonate gastropods that are distributed from marine to intertidal and terrestrial habitats (Romero et al. 2016). Therefore, the positive selection of *nad5* gene in *N.chinensis* may be the result of the adaptive evolution of freshwater environment. Moreover, divergent selection occurred at site 144 of *nad5*, where the amino acids Ala and Leu were identified in the freshwater *Novaculinachinensis* and seven marine lineages, respectively, indicating divergent evolution exists the family Pharidae. Divergent evolution is the process by which separate species with common ancestors evolve distinct features to adapt to their unique living environment, which is one of the important mechanisms for the formation of biodiversity (Gautam 2020). However, the evidence supporting the positive selection of individual nad5 sites is insufficient. To provide more robust statistical support for the differences in evolutionary adaptation between freshwater and seawater species, it is necessary to include more freshwater razor clam sequences.

## ﻿Conclusions

In summary, the mitogenomes of *S.rivularis* and *N.chinensis* were assembled using next-generation sequencing data, with the genomes measuring 17,159 bp and 15,957 bp, respectively. Both genomes consist of 12 protein-coding genes, 22 transfer RNA genes, and two ribosomal RNA genes. Among the published Pharidae mitogenomes, *N.chinensis* exhibits the smallest genome size but the highest AT content. The results of the phylogenetic analysis showed that *N.chinensis* and *Sinonovacula* (*S.constricta* + *S.rivularis*) were closely related and belonged to the family Pharidae. The gene order rearrangements in Solenoidea can be attributed to transposition and TDRL events. Moreover, the *nad5* genes carry a signal of positive selections in the foreground *N.chinensis*, which promotes the adaptation to freshwater environments. We also show that divergent evolution occurred at site 144 in the freshwater and marine lineages. Overall, this study provides further theoretical support for the phylogenetic relationship of Pharidae, and contributes to deepening the understanding of the mitogenomic adaptations of Pharidae.

## ﻿Additional information

### Data availability

All of the data that support the findings of this study are available in the main text.
